# Calcium and Vitamin D Supplementation for Prevention of Preeclampsia: A Systematic Review and Network Meta-Analysis

**DOI:** 10.3390/nu9101141

**Published:** 2017-10-18

**Authors:** Win Khaing, Sakda Arj-Ong Vallibhakara, Visasiri Tantrakul, Orawin Vallibhakara, Sasivimol Rattanasiri, Mark McEvoy, John Attia, Ammarin Thakkinstian

**Affiliations:** 1 Section for Clinical Epidemiology and Biostatistics, Faculty of Medicine, Ramathibodi Hospital, Mahidol University, 270 RAMA VI Road, Rachathevi, Bangkok 10400, Thailand; dr.winkhaing@gmail.com (W.K.); sasivimol.rat@mahidol.ac.th (S.R.); ammarin.tha@mahidol.ac.th (A.T.); 2 Department of Preventive and Social Medicine, University of Medicine, Mandalay 100102, Myanmar; 3 Sleep Disorder Center, Division of Pulmonary and Critical Care, Faculty of Medicine, Ramathibodi Hospital, Mahidol University, Bangkok 10400, Thailand; vtantrakul@gmail.com; 4 Department of Obstetrics and Gynecology, Faculty of Medicine, Ramathibodi Hospital, Mahidol University, Bangkok 10400, Thailand; orawinra38@gmail.com; 5 School of Medicine and Public Health, Faculty of Health and Medicine, The University of Newcastle, Callaghan, NSW 2308, Australia; mark.mcevoy@newcastle.edu.au (M.M.); john.attia@newcastle.edu.au (J.A.)

**Keywords:** calcium, network meta-analysis, gestational hypertension, preeclampsia, prevention, systematic review, vitamin D

## Abstract

Vitamin D supplementation effects with or without calcium in pregnancy for reducing risk of preeclampsia and gestational or pregnancy induced hypertension are controversial. Literature was systematically searched in Medline, Scopus and Cochrane databases from inception to July 2017. Only randomized controlled trials (RCTs) in English were selected if they had any pair of interventions (calcium, vitamin D, both, or placebo). Systematic review with two-step network-meta-analysis was used to indirectly estimate supplementary effects. Twenty-seven RCTs with 28,000 women were eligible. A direct meta-analysis suggested that calcium, vitamin D, and calcium plus vitamin D could lower risk of preeclampsia when compared to placebo with the pooled risk ratios (RRs) of 0.54 (0.41, 0.70), 0.47 (0.24, 0.89) and 0.50 (0.32, 0.78), respectively. Results of network meta-analysis were similar with the corresponding RRs of 0.49 (0.35, 0.69), 0.43 (0.17, 1.11), and 0.57 (0.30, 1.10), respectively. None of the controls were significant. Efficacy of supplementation, which was ranked by surface under cumulative ranking probabilities, were: vitamin D (47.4%), calcium (31.6%) and calcium plus vitamin D (19.6%), respectively. Calcium supplementation may be used for prevention for preeclampsia. Vitamin D might also worked well but further large scale RCTs are warranted to confirm our findings.

## 1. Introduction

Preeclampsia is a new onset of high blood pressure with proteinuria with/without end-organ or utero-placental dysfunction after 20 weeks of gestation. It is one of the major contributing causes of maternal-fetal morbidity and mortality worldwide [1]. Globally, 4.6% and 1.4% of all pregnancies developed preeclampsia and eclampsia, respectively [2]. The incidence in developed countries was approximately 3.4% [3], whereas it was varied from 1.8% to 16.7% in developing countries [4,5]. 

Approximately 10% to 15% of maternal death is directly associated with preeclampsia or eclampsia in low- and middle-income countries [6], whereas it was approximately one per 100,000 live births in developed countries [7]. It also related to life-threatening unfavorable outcomes in both mother (e.g., placental abruption, preterm delivery and hemolysis, elevated liver enzymes, and low platelets (HELLP) syndrome, etc.) and fetus (e.g., preterm birth, stillbirth, low birth weight, and small for gestational age, etc.) [8]. 

Previous evidence showed an inverse relationship between high blood pressure and calcium intake [9,10]. Numerous epidemiological and clinical studies [9,10,11], and later a series of systematic reviews [12,13,14,15] also demonstrated this association. Their results have suggested that calcium supplements (≥1 g/day) could lower the risk of preeclampsia [14]. As a result, the World Health Organization (WHO) has recommended to supplement calcium for pregnant women especially to high-risk population with a low calcium diet [16].

Vitamin D is involved in regulating bone metabolism, absorption of calcium and phosphate, and maintenance of muscle function [17]. Therefore, there might be a benefit of vitamin D supplementation in prevention of preeclampsia. However, systematic reviews [18,19] of randomized controlled trials (RCTs) did not show any benefit in prevention of preeclampsia, whereas other two systematic reviews [20,21] of observational studies did. These discrepancy results could be due to confounding bias in the latter or insufficient power in the former.

Although these pieces of evidence suggest benefits from both calcium and vitamin D supplements, it is still unclear which supplement or a combination of them is most beneficial for preventing preeclampsia and gestational hypertension (GH) or pregnancy induced hypertension (PIH). We therefore conducted a systematic review and a network meta-analysis (NMA) of RCTs with the aims of directly and indirectly comparing the effect of supplementations of calcium, vitamin D, both, and neither on preeclampsia and GH/PIH.

## 2. Materials and Methods 

A conventional pairwise meta-analysis can directly compare the efficacy or safety of exactly two treatments in head-to-head clinical trials that can comparative by use simple method of direct meta-analysis. However, in real practice, there are often many potential treatments for a single disease. NMA is an extension of standard pairwise meta-analysis that provides comprehensive comparative treatment effects by combining both direct and indirect evidence. Because of the possibility to combine evidence from different treatment comparisons, and because they can identify the single best available treatment for decision-making, NMA are becoming increasingly attractive to clinicians.

This systematic review was conducted according to the preferred reporting items for systematic reviews and meta-analyses (PRISMA), extension of network meta-analyses [22]. The review protocol has been registered with the international prospective register of systematic review (PROSPERO number CRD42015025389).

### 2.1. Search Strategy

Studies were located from Medline via PubMed, Scopus databases, and Cochrane library/Cochrane Central Register of Controlled Trials. The search terms and strategies were constructed based on PICO (i.e., patient, intervention, comparator, and outcome) as described in detail in Appendix A. These strategies were modified to suit each search engine where appropriate. 

Study identification was done in two phases. First, all previous systematic reviews of calcium and vitamin D supplementations in pregnant women published since inception of each database to July 2017 were identified. Then, only individual RCTs included in these previous reviews were selected. Second, all individual RCTs on the same topic published from inception to July 2017 were identified. The reference lists of the retrieved studies were also checked to identify more relevant publications. Where there were multiple publications from the same author(s) on the same topic, the most complete and recent study was included.

### 2.2. Study Selection

Identified studies from Medline, Scopus and Cochrane were imported into EndNote X7 and duplicate studies were removed. The selected studies were independently screened by title and abstract by two reviewers (W.K. and V.T.). Full texts were retrieved if decisions could not be reached from information provided in the abstract. Disagreements regarding selection were resolved by consensus or discussion with a third reviewer (S.A.V.). We contacted authors by email up to three times if data were insufficient. If there was no response after three attempts, then the study was excluded. 

All RCTs conducted in humans and published in English were included if they met all of the following criteria: (1) included pregnant women of any gestational age; (2) compared outcomes of interest between any pair of the following supplementation groups: calcium, vitamin D, combined calcium and vitamin D, and placebo/no supplementation; and (3) had at least one of the outcomes of interest including preeclampsia, eclampsia, GH or PIH. Studies were excluded from the review if they were crossover trials, included multiple pregnancies, or after three unsuccessful attempts requesting data from authors in the case of insufficient data. 

### 2.3. Interventions

Interventions were any of following supplements regardless of dosage and duration of supplements: Calcium, vitamin D, combined calcium and vitamin D. The control group could be placebo, a standard supplementation (e.g., folic acid), or no supplementation. 

### 2.4. Outcomes of Interest

The primary outcome of interest was preeclampsia, eclampsia, and GH/PIH, which were defined as per the original studies. Generally, preeclampsia was a new onset hypertension (i.e., systolic blood pressure ≥140 mmHg and/or diastolic blood pressure ≥90 mmHg for two occasions at least 4 h apart) and any of the following: proteinuria (dipstick urine 2+ or ≥300 mg/24 h), end-organ dysfunction, or utero-placental dysfunction after 20 weeks of gestation [23]. An early-onset preeclampsia occurred before 34 weeks of gestation, otherwise it was a late-onset preeclampsia. Eclampsia is a convulsive condition occurring in preeclampsia patients. GH/PIH is a new onset hypertension presenting after 20 weeks of gestational age without significant proteinuria [23].

### 2.5. Data Extraction

Two reviewers (W.K. and V.T.) independently extracted the relevant data (participants, interventions and outcome characteristics) and these were recorded using a standardized data extraction form (Appendix B). Co-variables such as mean age, gestational age at enrolment and delivery, gravida, parity, body mass index (BMI), smoking, diabetes mellitus, and duration of supplementation were also extracted. If duration of supplementation was not reported, it was calculated by subtracting gestational age at delivery with gestational age at initiating. If gestational age at delivery of that study was not reported, mean gestational age at delivery, i.e., 38 weeks, was used. Data entry, cleaning and checking were performed separately for each reviewer. The two datasets were compared and validated, and any disagreement resolved by consensus.

### 2.6. Risk of Bias Assessment

Study quality was independently assessed by two reviewers (W.K. and V.T.) using the Cochrane Collaboration tool for assessing risk of bias in RCTs version 5.1.0 [24], see Appendix C. The following seven domains were evaluated: selection bias (sequence generation and concealment), performance bias (blinding of participants and assessors), detection bias (blinding of outcome assessment), attrition bias (incomplete outcome data), selective outcome reporting, and other bias. Each item was classified as low, high, or an unclear risk of bias (if there was insufficient information).

### 2.7. Statistical Analysis

#### 2.7.1. Direct Meta-Analysis

For studies reporting frequency data of supplementation and preeclampsia, log risk ratio (RR) along with its variance and the 95% confidence interval (CI) were estimated for each study. The RRs were then directly pooled across studies using fixed-effect model (i.e., inverse variance method) if heterogeneity was absent, otherwise a random-effect model (i.e., DerSimonian and Laird method) was used.

Heterogeneity was assessed by Cochrane’s *Q* test and *I*^2^ statistic, respectively. If it was present (*p* < 0.1 or *I*^2^ ≥ 25%), a source of heterogeneity was explored by fitting characteristics of subjects (i.e., mean age, mean gestational age), clinical data (i.e., dosage, and duration of supplement), and methodological characteristics (i.e., definition of outcome measurements, setting of the study) in a meta-regression model one by one. Sensitivity analysis by excluding the outlier studies and/or a subgroup analysis according to that factor was performed.

#### 2.7.2. Network Meta-Analysis

Network meta-analysis was applied to indirectly compare effects of supplementation. A two-stage multivariate meta-analysis was applied as follows: Coefficients (i.e., *lnRR*) and variance-covariance of treatment comparisons were estimated for each study using a Poisson model. These parameters were then pooled across studies using a multivariate meta-analysis with maximum likelihood function [25]. Between-study variance and covariance of comparisons were considered using unstructured method. Effects between active versus active supplementation were then compared using a linear combination of the multivariate meta-analysis model. 

The inconsistency assumption (i.e., whether direct effects agree with the indirect effects) was checked and explored using a design-treatment interaction model, and an inconsistency factor (IF, i.e., *ln(RR_direct_)-ln(RR_indirect_))* was then estimated. Violation of consistency was assumed if the IF was significantly different from 0. All pairwise comparisons between direct and indirect effects, were estimated and displayed. In addition, small study effect for the whole network was assessed by constructing a comparison-adjusted funnel plot taking into account different comparisons [26]. This plots the difference of each study’s i observed *ln(RR)* of newer versus older supplement (y_iXY_) vs. the comparison’s mean ln(RR, μ_XY_) against its variance. Supplementations were coded from older to newer as 1, 2, 3, 4 for placebo, calcium, vitamin D, and calcium plus vitamin D, respectively. In the absence of small-study effects, we expected the studies to form an inverted funnel centered at zero, i.e., the comparison-adjusted funnel plot should be symmetrical around the zero line. Finally, a predictive probability of best intervention was estimated using surface under a cumulative ranking curve (SUCRA). Efficacy of supplementation was then ranked by predicting probability.

All analyses were performed using STATA version 14.2 [27]. *P*-values < 0.05 were considered as statistically significant, except for the test of heterogeneity where *p* < 0.10 was used. 

## 3. Results

### 3.1. Study Selection and Characteristics

**Figure 1:**
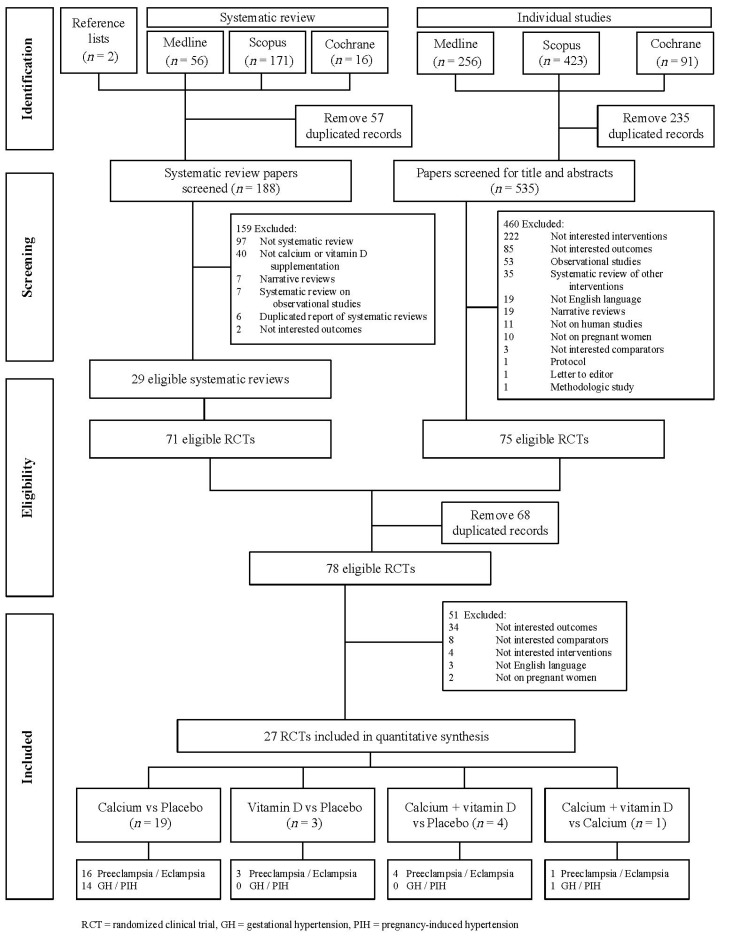
Flow of selection of studies.

The schema for selection of studies is displayed in Figure 1. Searching for previous systematic reviews identified 188 review studies. Among these, 159 review studies were excluded for reasons describe in Figure 1, leaving 29 review studies with individual 71 RCTs that were eligible for further assessment. In searching for individual studies, 535 studies were identified for screening titles and abstracts. Among these, 460 studies were excluded leaving 75 individual RCTs that met inclusion criteria for further assessment. After removing duplicates with searching from systematic reviews, 78 RCTs were eligible for assessing full-text. Of these, only 27 RCTs studies met our inclusion criteria and were considered for quantitative synthesis. Among these, 12, 3, and 12 RCTs studies reported preeclampsia, GH or PIH, and both outcomes, respectively. 

The characteristics of the 27 RCTs are described in Table 1. Among these, 19 studies (*n* = 26,299) compared calcium vs. placebo [11,28,29,30,31,32,33,34,35,36,37,38,39,40,41,42,43,44,45], three studies (*n* = 357) [46,47,48] compared vitamin D vs. placebo, four studies (*n* = 1169) [49,50,51,52] compared calcium plus vitamin D vs. placebo, and one study (*n* = 175) [53] compared calcium plus vitamin D vs. calcium. 

Cross-tabulated data for these interventions with preeclampsia and GH/PIH are described in Tables S1 and S2. Individual sample sizes ranged from 30 to 9178 with a median of 178. The types of pregnant women varied, 48.2% (13/27) of RCTs studies in low risk pregnancies and 51.9% (14/27) RCTs studies in high risk pregnancies, e.g., adolescent pregnancy, elderly pregnancy, and nulliparity. The mean age ranged from 16 to 37.2 years, and mean gestational age at enrolment and at delivery ranged from 14.2 to 29.7 and 37.4 to 39.1 weeks, respectively. 

### 3.2. Risk of Bias Assessment

Risk of bias assessment was performed for each RCT (Table S3) and summarized in Figure S1. Among 27 RCTs, three studies [29,37,38] were conference abstracts, thus the risk of bias could not be assessed because authors did not publish full articles. In the remaining 24 studies, sequence generation was clearly described in 17 trials (70.8%), whereas five trials (20.8%) were unclear. Allocation concealment was adequately performed in 13 trials (54.2%). Most studies (16/24) reported about blinding of participants and blinding of outcome assessors, whereas 12 trials (50%) reported incomplete outcome data. Haft of RCTs (12/24) had low risk of bias for selective outcome reports, and intention-to-treat (ITT) analysis was used in 15/24 trials.

### 3.3. Direct Meta-Analysis

#### 3.3.1. Preeclampsia

Direct comparisons with calcium vs. placebo, vitamin D vs. placebo and calcium plus vitamin D vs. placebo in preeclampsia were pooled across 16 RCTs (*n* = 12,876 vs. 13,060), three RCTs (*n* = 203 vs. 154) and four RCTs (*n* = 584 vs. 585), respectively. These corresponding pooled effects were 0.54 (95% CI: 0.41, 0.70), 0.47 (95% CI: 0.24, 0.89) and 0.50 (95% CI: 0.32, 0.78), respectively (see Figure S2a–e). This indicated that calcium, vitamin D and calcium plus vitamin D supplementations could reduce preeclampsia risk by approximately 46%, 53% and 50% when compared with placebo, respectively.

Sources of heterogeneity for the pooled calcium vs. placebo effect were explored using a meta-regression, as mentioned in the Materials and Methods Section. Only type of pregnancy (low versus high risk pregnancy) and duration of calcium supplementation (>18 vs. ≤18 weeks) could reduce the degree of heterogeneity from 72.6% to 61.84% and 61.51%, respectively. Subgroup analysis was therefore performed accordingly. The protective effect of calcium supplementation was greater in high risk pregnancies than low risk pregnancies with a pooled RR of 0.42 (95% CI: 0.28, 0.64) and 0.69 (95% CI: 0.52, 0.91), respectively (see Figure S2a). The calcium supplement effect was also higher in pregnant women whose supplement durations were 18 weeks or shorter but not for longer than 18 weeks with the pooled RRs of 0.36 (95% CI: 0.23, 0.54) and 0.69 (95% CI: 0.53, 0.91), see Figure S2b. In addition, subgroup analysis by country of setting (developing and developed countries) showed significant preventive effects of calcium in developing countries but not for developed countries with the pooled RR of 0.50 (95% CI: 0.35, 0.70) and 0.52 (95% CI: 0.26, 1.07), respectively (see Figure S2c).

#### 3.3.2. GH/PIH

Fourteen RCTs compared effects of calcium vs. placebo on risk of GH or PIH (*n* = 12,394 vs. 12,519), but only one RCT compared calcium plus vitamin D versus calcium (see Table S2). Effects of calcium compared to placebo were heterogeneous (*I*^2^ = 64.8%) with the pooled RR of 0.77 (95% CI: 0.65, 0.92). Sources of heterogeneity were next explored, as none were identified as a source of heterogeneity.

### 3.4. Network Meta-Analysis

Data from 24 RCTs were used in a network meta-analysis (see Table S1). Only studies on preeclampsia were included in indirect comparison, because there was a lack of RCT studies which reported on effect of vitamin D alone or combination with calcium for GH or PIH causing insufficient data for pooling.

All interventions were mapped in a network plot (Figure S3). The size of each node was proportional to the number of included studies, whereas the edge of each comparison was weighted by the number of pregnant women for that comparison. Two indirect comparisons were performed by “borrowing” data from common comparators in the network, i.e., vitamin D vs. calcium and calcium plus vitamin D vs. vitamin D, respectively. 

**Figure 2:**
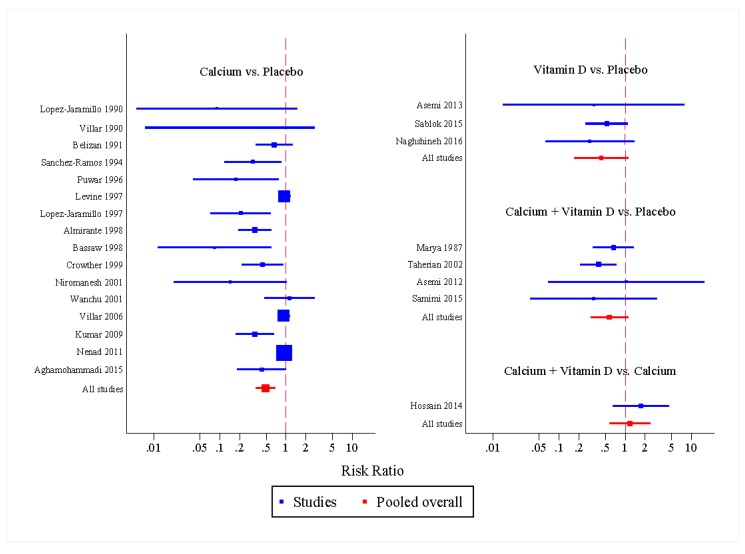
Forest plot of intervention effects compared to placebo on preeclampsia: a network meta-analysis.

The network meta-analysis indicated calcium significantly reduced risk of preeclampsia by 51% when calcium was used as prophylaxis when compared with placebo (RR of 0.49, 95% CI: 0.35, 0.69). Vitamin D alone also seemed to be as effective as calcium alone. It could reduce risk of preeclampsia by 57% when compared to placebo with a pooled RR of 0.43 (95% CI: 0.17, 1.11), but it was not statistically significant. When compared indirectly with placebo or no supplement, calcium plus vitamin D showed non-statistically significant reduction in preeclampsia (RR 0.57, 95% CI: 0.30, 1.10) (Figure 2). 

All multiple comparisons were further estimated (Table 2) suggesting vitamin D alone seemed to be better than calcium supplement alone but this was not statistically significantly different with pooled RRs of 0.89 (95% CI: 0.33, 2.41). Combination of vitamin D with calcium did not seem effective on prevention of preeclampsia when compared with calcium alone (RR 1.18, 95% CI: 0.58, 2.37) or vitamin D alone (RR 1.33, 95% CI: 0.42, 4.18) (Table 2).

Ranking of all interventions was performed using the method of SUCRA, a summary statistic for the cumulative ranking and probability of ranking (Table 2 and Figure S4). SUCRA ranges from 0 to 1, where 1 reflects the best treatment with no uncertainty and 0 reflects the worst treatment with no uncertainty. Our findings suggested that vitamin D was the first rank, followed by calcium, and then calcium plus vitamin D. The estimated ranking probabilities for these corresponding supplements were 47.4%, 31.6%, and 19.6%, respectively. Furthermore, a design-by-treatment inconsistency model was applied which suggested that there was no evidence of inconsistency between direct and indirect effects (Chi-square test = 0.42, *p* = 0.517). In addition, transitivity was further indirectly assessed by exploring and comparing characteristics of pregnant women across four supplement arms (i.e., calcium versus placebo, vitamin D versus placebo, calcium plus vitamin D versus placebo, and calcium plus vitamin D versus calcium (see Table S4)). This indicated that their characteristics were not much different; for instance, mean gestational age at initiating supplementation raged from 20 to 21.8 weeks, mean maternal age ranged from 21.2 to 25.6 years, and mean gestational age at delivery ranged from 37.4 to 38.8. However, mean BMI was quite different, which ranged from 23.6 to 30.8 kg/m^2^. 

A comparison-adjusted funnel plot was constructed indicating asymmetry of the funnel, i.e., there might be small study-effects, particularly from studies with calcium versus placebo (Figure S5). Sample sizes of all studies ranged from 30 to 9178, with a median of 178. A sensitivity analysis was then performed by excluding studies whose sample sizes were small, i.e., those that were in the first quartile of smallest sample size. Three RCTs [37,39,42] comparing calcium vs. placebo (*n* = 49), one [46] comparing vitamin D vs. placebo (*n* = 54) and two [49,51] comparing calcium plus vitamin D vs. placebo (*n* = 109) were excluded. However, the results did not show much difference from pooling all trials. The pooled RRs were 0.54 (95% CI: 0.38, 0.78) and 0.44 (95% CI: 0.17, 1.18) for calcium vs. placebo and vitamin D vs. placebo, respectively. Thus, there was little effect of small study influence on our pooled estimates.

Finally, numbers needed to treat (NNT) for calcium vs. placebo (16 RCTs, *n* = 12,876 vs. 13,060), vitamin D vs. placebo (3 RCTs, *n* = 203 vs. 154) and calcium plus vitamin D vs. placebo (4 RCTs, *n* = 584 vs. 585) in preeclampsia were estimated without subgrouping. We found that 19 (95% CI: 15, 32) pregnant women were needed to receive supplements with calcium to prevent one episode of preeclampsia. However, the NNTs for supplement with vitamin D and calcium plus vitamin D ranged from benefits to harms, with the estimated NNTs of 17 (95% CI: −89, 12) and 23 (95% CI: −98, 14), respectively.

## 4. Discussion

We have performed a systematic review and network meta-analysis of calcium and vitamin D supplementation effects on preeclampsia and GH/PIH risk. Our finding from direct meta-analysis suggested that calcium supplementation could significantly reduce the risk of preeclampsia and GH/PIH by approximately 50% and 25%, respectively, when compared with placebo. Supplementation appeared more effective in high risk pregnancies than in low risk pregnancies, and in those who consumed the supplement for 18 weeks or shorter duration. The network meta-analysis indicated the best supplementation for lowering preeclampsia was vitamin D, followed by calcium and calcium plus vitamin D. The NNTs for these corresponding supplements would be 17, 19, and 23, respectively; however, only NNT for calcium supplement was beneficial, whereas the rest supplements could be either beneficial or harmful.

Although our diagnostics do not indicate any heterogeneity or little effect of small study effects, these results are based on very small numbers of participants. Our results are consistent with the updated Cochrane review [15], which found significant preventive effect of calcium supplementation on preeclampsia, especially in high risk women. Tang et al. also found the same effect of calcium supplementation in high risk, but not for low risk pregnancy [54]. In addition, effect of calcium supplement was significantly benefit in developing but not for developed countries, which was consistent to Imdad et al. [55], who found benefit of calcium supplement in developing countries where calcium intake was low. Therefore, the WHO has recommended prescribing calcium supplementation in routine antenatal care to those high risk pregnant women with low calcium intake for prevention of preeclampsia.

The findings of vitamin D and calcium plus vitamin D supplementation are also similar with the latest Cochrane review [19]. The effects of these supplementations might reduce the risk of preeclampsia, but further better-quality RCTs are still needed to confirm the effects.

### 4.1. Strengths and Limitations

Our study had a number of strengths. In comparison with earlier systematic reviews of observational studies, our meta-analysis included only RCTs, thus selection bias and confounding biases should be minimized. We compared effects of all supplementations on preeclampsia using network meta-analysis to indirectly compare efficacy between supplementations by borrowing data from common comparators. Neither publication bias nor inconsistency was detected. A ranking of interventions with their NNTs has also been calculated.

However, our study also had some limitations. Some relevant studies might be missing from our pooling because neither grey literature databases were used for identifying studies, nor non-English studies were considered. The number of included studies for vitamin D and calcium plus vitamin D were very small, and thus estimation of supplementation effects were imprecise. Different dosages of supplementations had been used, and given the small number of included studies we were unable to tease out a dosage effect. Although transitivity could not be directly assessed with aggregated meta-data, our study indirectly assessed transitivity assumption by extracting patient and methodological characteristics of included studies (i.e., dosage, duration of use, gestational age at start supplementation, gestational age at enrolment and delivery).

### 4.2. Summary of Evidence

Our meta-analysis has advantages over previous systematic reviews by integrating both direct and indirect comparisons of calcium, vitamin D, and calcium plus vitamin D supplementation in the entire network approach. Effects of calcium supplement on preeclampsia were robust and consistent for both direct and indirect meta-analyses. In addition, these effects were more beneficial if duration of receiving calcium supplementation was 18 weeks or shorter. As for our data, most included studies had mean gestational age at delivery of 38 weeks. This implies that calcium supplementation should be initiated at about 20 weeks or later. As for the pathophysiology of preeclampsia, abnormality of placenta might release secreted factors in mother’s circulations, which resulted in clinical manifestation of preeclampsia occurring during 20 weeks of gestational age or after [56,57,58]. Increasing calcium concentration might play a role in decreasing smooth muscle contractility and increasing vasodilation, thus lowering risk of preeclampsia [9,59].

Until now, there has been no RCTs directly assessing the efficacy of supplementation on preeclampsia comparing calcium vs. vitamin D, and calcium plus vitamin D vs. vitamin D, but our network meta-analysis extrapolated these results. Vitamin D might be preferred for preventing preeclampsia. Possible explanations for this result might be as follows: First, adequate vitamin D intake might maintain calcium homeostasis, which in turn has an inverse relationship with blood pressure [10], or might directly suppress vascular smooth muscle cell proliferation [60]. Second, vitamin D might be a potent endocrine suppressor of renin biosynthesis and regulate the renin-angiotensin system, which plays a critical role in the regulation of blood pressure [60]. Third, vitamin D might have immune-modulatory effect by balancing T helper cells [61]. 

Although supplementation of vitamin D with/without calcium ranked higher than calcium supplementation alone, this needs to be confirmed in head to head trials. However, the evidence was not enough to make a conclusive statement for using vitamin D in both developed and developing countries. If proven, applying this in routine care of pregnancy might be more difficult particularly in developing countries because of its greater investigation and prophylaxis cost when compared to calcium supplements. This suggests that calcium supplementation alone may remain the standard of choice for the prevention of preeclampsia and also in term of safeties when accessibility to addition of vitamin D to calcium is limited. 

Currently, with existing evidence, vitamin D is still far from being recommended for prevention of preeclampsia according to Heaney’s criteria [62]. Further research should focus on the recommended daily allowance of vitamin D for pregnant women, minimally clinical effective dosage of vitamin D, safety of vitamin D with different dosages, timing of initiation of supplementation in pregnancy, supplementation regimen (daily or weekly or single dose), supplementation alone or in combination with other nutrients, and to which type of pregnancy (low or high risk).

## 5. Conclusions

Our evidence suggests that calcium supplementation could reduce risk of preeclampsia. Vitamin D supplementation might also be beneficial, but this was based on evidence from a small number of studies examining vitamin D with short term assessment. Therefore, larger, well-designed RCTs are still required to determine the efficacy of vitamin D supplementation alone or in combination with calcium to reduce the risk of preeclampsia. Conversely, this network meta-analysis needs to be updated once more RCTs of vitamin D supplementation are available.

## Figures and Tables

**Table 1:** Characteristics of included studies.

Author (Year)	Country	Outcome	Study Period (Months)	Type of Pregnancy	*n* Control	*n* Intervention	Supplement Started GA (Weeks)	Mean Age (Years)	Mean GA at Enrolment (Weeks)	Mean GA at Delivery (Weeks)	SBP (mmHg)	DBP (mmHg)	BMI (kg/m^2^)	Weight Gain (g/Week)	Nulliparity (%)
***Calcium vs. Placebo***															
Aghamohammadi (2015) [28]	Iran	PE	-	High Risk Women	40	40	<20 weeks	37.15	-	-	-	-	26.8	-	-
Almirante (1998) [29]	Philippines	PE	-	High Risk Women	210	212	<20 weeks	-	18.00	-	-	-	-	-	100.00
Bassaw (1998) [30]	Bangladesh	Both	36	Low Risk Women	250	81	<20 weeks	27	-	38.6	-	-	-	-	-
Belizan (1991) [31]	Argentina	Both	33	High Risk Women	588	579	≥20 weeks	23.70	20.80	-	103.95	66.45	-	-	100.00
Crowther (1999) [32]	Australian	Both	53	Low Risk Women	229	227	≥20 weeks	24.70	18.37	-	115.80	68.20	26.60	-	100.00
Kumar (2009) [33]	New Delhi	PE	36	Low Risk Women	251	273	<20 weeks	21.85	17.83	38.44	113.19	74.00	23.35	-	-
Levine (1997) [34]	US	Both	36	Low Risk Women	2294	2295	<20 weeks	21.00	17.15	38.90	106.50	59.70	-	-	100.00
Lopez-Jaramillo (1997) [35]	Ecuador	PE	56	High Risk Women	135	125	≥20 weeks	15.99	20.00	39.13	-	-	-	414.19	100.00
Lopez-Jaramillo (1990) [37]	Ecuador	Both	30	Low Risk Women	34	22	≥20 weeks	19.4	-	-	-	-	-	-	100.00
Lopez-Jaramillo (1989) [36]	Ecuador	GH/PIH	30	Low Risk Women	43	49	≥20 weeks	18.47	23.00	-	-	-	-	430.80	100.00
Nenad (2011) [38]	Serbia	Both	-	Low Risk Women	4588	4590	<20 weeks	-	18.50	-	-	-	-	-	100.00
Niromanesh (2001) [39]	Iran	Both	-	High Risk Women	15	15	≥20 weeks	23.15	29.70	38.60	-	-	-	-	-
Puwar (1996) [40]	India	Both	15	Low Risk Women	93	97	≥20 weeks	21.93	18.07	37.50	103.02	63.32	-	-	100.00
Rogers (1999) [41]	Hong Kong	GH/PIH	30	High Risk Women	75	144	≥20 weeks	27.31	21.67	38.9	-	-	-	-	100.00
Sanchez-Ramos (1994) [42]	Florida	Both	55	High Risk Women	34	29	≥20 weeks	18.38	24.44	-	113.50	64.01	-	-	100.00
Villar (1987) [11]	Baltimore, Argentina	GH/PIH	36	Low Risk Women	27	25	≥20 weeks	21.10	-	-	-	-	-	388.2	100.00
Villar (1990) [44]	Baltimore	Both	36	High Risk Women	88	90	≥20 weeks	16.25	23.55	38.55	102.75	61.10	-	-	85.26
Villar (2006) [43]	Argentina, Egypt, India, Peru, South Africa, Vietnam	Both	21	Low Risk Women	4161	4151	<20 weeks	22.65	15.10	-	105.05	60.80	21.90	-	100.00
Wanchu (2001) [45]	India	PE	-	High Risk Women	50	50	≥20 weeks	-	14.2	-	111.57	72.45	-	-	100.00
***Vitamin D vs. Placebo***															
Asemi (2013) [46]	Iran	PE	4	High Risk Women	27	27	≥20 weeks	17.44	26	-	-	-	30.8	-	-
Naghshineh (2016) [47]	Iran	PE	5	High Risk Women	70	68	<20 weeks	25	-	37.4	-	-	-	-	100.00
Sablok (2015) [48]	India	PE	36	High Risk Women	57	108	<20 weeks	-	-	-	-	-	-	-	100.00
***Calcium plus Vitamin D vs. Placebo***															
Asemi (2012) [49]	Pakistan	PE	11	High Risk Women	25	24	≥20 weeks	24.9	-	-	-	-	27.58	-	100.00
Marya (1987) [50]	India	PE	-	Low Risk Women	200	200	≥20 weeks	-	22.00	-	-	-	-	-	-
Taherian (2002) [52]	Iran	PE	36	Low Risk Women	330	330	≥20 weeks	21.55	20.00	38.80	97.25	57.88	22.55	10.25 *	-
Samimi (2015) [51]	Iran	PE	6	High Risk Women	30	30	≥20 weeks	27.2	-	-	111.7	72.4	26.5	-	-
***Calcium plus Vitamin D vs. Calcium***															
Hossain (2014) [53]	Pakistan	Both	21	Low Risk Women	89	86	≥20 weeks	25.57	20.00	37.61	-	-	23.64	-	-

*n* = number of subjects, GA = Gestational Age (weeks), SBP = Mean Systolic Blood Pressure (mmHg), DBP = Mean Diastolic Blood Pressure (mmHg), BMI = Mean Body Mass Index (kg/m^2^), PE = Preeclampsia only, GH/PIH = Gestational Hypertension or Pregnancy Induced Hypertension only, Both = Both PE or GH/PIH, * Mean weight gain in kg.

**Table 2:** Estimation of multiple supplementation effects on preeclampsia.

Intervention	Calcium	Vitamin D	Calcium + Vitamin D
**Calcium**	0.49 (0.35 0.69) * {66.1, 31.6}	0.89 (0.33, 2.41) *^†^	1.18 (0.58, 2.37) *^‡^
**Vitamin D**		0.43 (0.17, 1.11) ^†^ {70.7, 47.4}	1.33 (0.42, 4.18) ^†‡^
**Calcium + Vitamin D**			0.57 (0.30, 1.10) ^‡^ {52.2, 19.6}

Values are expressed as pooled RR along with 95% CIs in round parentheses; on diagonal cells comparing supplement vs. placebo, off the diagonal cells comparing column vs. row supplements; values < 1 indicates that the intervention listed in the column is more effective than the one in the row; Values in the diagonal in curly parentheses indicate surface under the cumulative ranking curve and the probability of being the best treatment. The larger is the surface under the cumulative ranking curve or probability of being the best treatment, the better is the treatment. * Calcium vs. Placebo: 16 RCTs, *n* = 12,876 vs. 13,060, number of PE cases = 722 vs. 931; ^†^ Vitamin D vs. Placebo: 3 RCTs, *n* = 203 vs. 154, number of PE cases = 20 vs. 14; ^‡^ Calcium + Vitamin D vs. Placebo: 4 RCTs, *n* = 584 vs. 585, number of PE cases = 27 vs. 55; *^‡^ Calcium + Vitamin D vs. Calcium: 1 RCT, *n* = 89 vs. 86, number of PE cases = 10 vs. 6; *^†^ Calcium vs. Vitamin D: 19 RCTs, *n* = 25,936 vs. 357, number of PE cases = 722 vs. 14; ^†‡^ Calcium + Vitamin D vs. Vitamin D: 7 RCTs, *n* = 1169 vs. 357, number of PE cases = 55 vs. 14.

## Supplementary Materials

The following are available online at www.mdpi.com/2072-6643/9/10/1141/s1, Table S1: Direct comparisons between supplementations on preeclampsia; Table S2: Direct comparisons between supplementations on gestational hypertension or pregnancy induced hypertension; Table S3: Risk of Bias Assessment; Table S4: Patient and methodological characteristics of included studies by treatment; Figure S1: Risk of bias assessment; Figure S2: Direct comparisons of supplementations on preeclampsia (a) Calcium vs. placebo by participants (b) Calcium vs. placebo by duration of calcium supplementation (c) Calcium vs. placebo by country setting (d) Vitamin D vs. placebo (e) Calcium plus vitamin D vs. placebo; Figure S3: Network of all possible interventions for prevention of preeclampsia; Figure S4: Rankogram of calcium, vitamin D, calcium plus vitamin D and placebo effects on preeclampsia; Figure S5: Comparison-adjusted funnel plot for the network meta-analysis.

## Appendix A. Search Terms and Search Strategy

### Appendix A.1. PubMed Database

(((((((((((calcium supplement*)) OR (“calcium carbonate”)) OR (“calcium gluconate”)) OR (“calcium acetate”)) OR (“calcium citrate”)) OR (“calcium lactate”))) OR ((((“vit* D supplement*”)) OR (“cholecalciferol”)) OR (“ergocalciferol”)))) AND (((“Pregnant Women”[Mesh])) OR (“Pregnancy”[Mesh]))) AND ((((((((((“Pre-Eclampsia”[Mesh])) OR (“Eclampsia”[Mesh])) OR (preeclampsia)) OR (“gestational hypertension”)) OR (“gestational hypertensive disorder”)) OR (“hypertensive disorder during pregnancy”)) OR (“pregnancy induced hypertension”)) OR (PIH)) OR (“pre-clamptic toxaemia”))

### Appendix A.2. Scopus Database

((TITLE-ABS-KEY (“calcium supplement*” OR “calcium carbonate” OR “calcium gluconate” OR “calcium acetate” OR “calcium citrate” OR “calcium lactate”)) OR (TITLE-ABS-KEY (“vit* D supplement*” OR “cholecalciferol” OR “ergocalciferol”))) AND (TITLE-ABS-KEY (“pre-Eclampsia” OR eclampsia OR preeclampsia OR “gestational hypertension” OR “gestational hypertensive disorder” OR “hypertensive disorder during pregnancy” OR “pregnancy induced hypertension” OR pih OR “pre-clamptic toxaemia”)) AND (TITLE-ABS-KEY (“pregnant women” OR “pregnancy”)).

**Table A1:** PICO Searching.

	Domain	Terms
1	Pregnancy	“pregnancy”[Mesh] ◈
2		“pregnant women”
3		1 OR 2
4	Calcium supplementation	calcium supplement*
5		“calcium carbonate”
6		“calcium gluconate”
7		“calcium acetate”
8		“calcium citrate”
9		“calcium lactate”
10		4 OR 5 OR 6 OR 7 OR 8 OR 9
11	Vitamin D supplementation	“vit* D supplement*”
12		“cholecalciferol”
13		“ergocalciferol”
14		11 OR 12 OR 13
15	Calcium or Vitamin D	10 OR 14
16	Preeclampsia	“pre-eclampsia”[Mesh] ◈
17		“eclampsia”[Mesh] ◈
18		“preeclampsia”
19		“gestational hypertension”
20		“gestational hypertensive disorder”
21		“hypertensive disorder during pregnancy”
22		“pregnancy induced hypertension”
23		PIH
24		“pre-eclamptic toxaemia”
25		16 OR 17 OR 18 OR 19 OR 20 OR 21 OR 22 OR 23 OR 24
26		3 AND 15 AND 25
27		systematic[sb] AND 26 ◈

◈ Option for Medline.

## Appendix B

### Appendix B.1. Data Extraction Form

**Table A2:** General information of the study.

Study ID	………………………………………………………………………		
Reviewer	………………………………………………………………………		
Date of review	(DD/MM/YYYY) ………………………………….………………		
Study title	………………………………………………………………………		
First Authors	………………………………………………………………………		
Journal	………………………………………………	Year	…………

**Table A3:** Study Setting

Setting	☐ 1. Hospital Based	☐ 2. Community Based
Country of study	…………………………………………………………………	

**Table A4:** General Characteristics of study.

Study Design	☐ 1. Randomized Controlled Trial	☐ 2. Quasi-Experimental Design
Period of the study	……………………………………………… months	

**Table A5:** Participants.

**Pregnant Women**	**☐ 1. Low Risk Women** **☐ 2. High Risk Women**

**Table A6:** Intervention.

**Calcium Supplementation**			
☐ 1. Yes ☐ 2. No			
Form	☐ 1. Calcium carbonate ☐ 2. Calcium gluconate ☐ 3. Calcium acetate ☐ 4. Calcium citrate ☐ 5. Calcium lactate ☐ 6. Not specified ☐ 7. Other ……………………………		
Dosage	…………………… g	Duration	………… weeks
Timing	☐ 1. Single Dose ☐ 2. Daily ☐ 3. Weekly ☐ 4. Monthly ☐ 5. Other ………………..		
Started at	☐ 1. First Trimester (0 to 13 Weeks) ☐ 2. Second Trimester (14 to 26 Weeks) ☐ 3. Third Trimester (27 to 40 Weeks) ☐ 4. No mention		
Co-Supplement	☐ 1. Yes, specify…………………… ☐ 2. No		
**Vitamin D Supplementation**			
☐ 1. Yes ☐ 2. No			
Form	☐ 1. Ergocalciferol ☐ 2 .Cholecalciferol ☐ 3. Not specified ☐ 4. Other ……………………………		
Dosage	…………………… IU	Duration	………… weeks
Timing	☐ 1. Single Dose ☐ 2. Daily ☐ 3. Weekly ☐ 4. Monthly ☐ 5. Other ………………..		
Started at	☐ 1. First Trimester (0 to 13 Weeks) ☐ 2. Second Trimester (14 to 26 Weeks) ☐ 3. Third Trimester (27 to 40 Weeks) ☐ 4. No mention		
Co-Supplement	☐ 1. Yes, specify…………………… ☐ 2. No		
**Calcium plus Vitamin D Supplementation**			
☐ 1. Yes ☐ 2. No			
Calcium Form	☐ 1. Calcium carbonate ☐ 2. Calcium gluconate ☐ 3. Calcium acetate ☐ 4. Calcium citrate ☐ 5. Calcium lactate ☐ 6. Not specified ☐ 7. Other ……………………………		
Dosage	…………………… g	Duration	………… weeks
Timing	☐ 1. Single Dose ☐ 2. Daily ☐ 3. Weekly ☐ 4. Monthly ☐ 5. Other ………………..		
Vit D Form	☐ 1. Ergocalciferol ☐ 2 .Cholecalciferol ☐ 3. Not specified ☐ 4. Other ……………………………		
Dosage	…………………… IU	Duration	………… weeks
Timing	☐ 1. Single Dose ☐ 2. Daily ☐ 3. Weekly ☐ 4. Monthly ☐ 5. Other ………………..		
Started at	☐ 1. First Trimester (0 to 13 Weeks) ☐ 2. Second Trimester (14 to 26 Weeks) ☐ 3. Third Trimester (27 to 40 Weeks) ☐ 4. No mention		
Co-Supplement	☐ 1. Yes, specify…………………… ☐ 2. No		

**Table A7:** General baseline characteristics of participants.

Characteristics	Intervention *n* =	Control *n* =	Total *n* =
Mean Age (year)			
Mean Gestation age at enrolment (week) (mean, SD)			
SBP (mean, SD)			
DBP (mean, SD)			
Abnormal Proteinuria (%)			
BMI (mean, SD)			
Primigravida (%)			
Nulliparous (%)			
Gestational Diabetes Mellitus (%)			
Smoking (%)			
Mean total weight gain (mean, SD)			
% Withdraw (lost FU)			
Mean gestational age at delivery (mean, SD)			
Mean baseline calcium level (mean, SD)			
Mean baseline vitamin D level (mean, SD)			

**Table A8:** Type of Outcomes and Definitions.

**Preeclampsia**	☐ 1. Yes ☐ 2. No	
	☐ Preeclampsia	☐ SBP ≥ 140 mmHg, DBP ≥ 90 mmHg and Proteinuria >2+
	☐ Severe preeclampsia	☐ SBP ≥ 160 mmHg, DBP ≥ 1100 mmHg and Proteinuria >2+
	☐ Early onset preeclampsia	☐ preeclampsia occur <34 weeks’ gestation
	☐ Late onset preeclampsia	☐ preeclampsia occur ≥34 weeks’ gestation
**Eclampsia**	☐ 1. Yes ☐ 2. No	
**GH or PIH**	☐ 1. Yes ☐ 2. No SBP ≥ 140 mmHg or DBP ≥ 90 mmHg	

### Appendix B.2. Intervention and Outcomes

**Table A9:** Dichotomous outcomes for Preeclampsia.

Treatment	Preeclampsia			
	Yes	No	RR/OR	95% CI
Calcium				
Vitamin D				
Calcium + Vit D				
Placebo				

**Table A10:** Dichotomous outcomes for Eclampsia.

Treatment	Eclampsia			
	Yes	No	RR/OR	95% CI
Calcium				
Vitamin D				
Calcium + Vit D				
Placebo				

**Table A11:** Dichotomous outcomes for GH/PIH.

Treatment	GH/PIH			
	Yes	No	RR/OR	95% CI
Calcium				
Vitamin D				
Calcium + Vit D				
Placebo				

## Appendix C. Cochrane “Risk of Bias” Assessment

**Table A12:** Risk of bias.

	Low (2)	High (1)	Unclear (0)	Comment
Adequate sequence generation	☐	☐	☐	
Allocation concealment	☐	☐	☐	
Blinding of participants and personnel	☐	☐	☐	
Blinding of outcome assessment	☐	☐	☐	
Incomplete outcome data addressed	☐	☐	☐	
Selective outcome reporting	☐	☐	☐	
Other sources of bias	☐	☐	☐	

### Criteria for Judging Risk of Bias in the “Risk of Bias Assessment” Tool

Higgins JPT, Green S, editors. Cochrane Handbook for Systematic Reviews of Interventions Version 5.1.0 [updated March 2011]. The Cochrane Collaboration, 2011. Available from www.cochrane-handbook.org.

**Table A13:** Random Sequence Generation.

Selection Bias (Biased Allocation to Interventions) Due to Inadequate Generation of a Randomized Sequence	
Criteria for judgment of “Low risk” of bias	Randomization was performed using any of following methods: Using a random number table; Simple or block or stratified randomization by using a computer random number generator with or without detailed description of generation process; Tossing Coin; Shuffling cards or envelopes; Throwing dice; Drawing of lots;
Criteria for judgment of “High risk” of bias	Systematic, non-random sequence generation was performed using any of the follow methods: Odd or even sequence generated by birth date; Sequence generated by some rule based on date (or day) of admission; Sequence generated by some rule based on hospital or clinic record number. Categorized with non-random approach using any of the following methods: by judgement of the clinician; by preference of the participant; based on the results of a laboratory test or a series of tests; by availability of the intervention.
Criteria for judgment of “Unclear risk” of bias	Insufficient information about the sequence generation process to permit judgement of ‘Low risk’ or ‘High risk’.

**Table A14:** Allocation Concealment.

Selection Bias (Biased Allocation to Interventions) Due to Inadequate Concealment of Allocations Prior to Assignment	
Criteria for judgment of “Low risk” of bias	If any of following was applied or mentioned Central allocation (including telephone, web-based and pharmacy-controlled randomization); Sequentially numbered drug containers of identical appearance; Sequentially numbered, opaque, sealed envelopes.
Criteria for judgment of “High risk” of bias	Authors used any of following Using an open random allocation schedule (e.g., a list of random numbers); Assignment envelopes were used without appropriate safeguards (e.g., if envelopes were unsealed or non-opaque or not sequentially numbered); Alternation or rotation; Date of birth; Case record number; Any other explicitly unconcealed procedure.
Criteria for judgment of “Unclear risk” of bias	Insufficient information to permit judgement of ‘Low risk’ or ‘High risk’. This is usually the case if the method of concealment is not described or not described in sufficient detail to allow a definite judgement–for example if the use of assignment envelopes is described, but it remains unclear whether envelopes were sequentially numbered, opaque and sealed

**Table A15:** Blinding of Participants and Personnel.

Performance Bias Due to Knowledge of the Allocated Interventions by Participants and Personnel during the Study	
Criteria for judgment of “Low risk” of bias	Any one of the following: Blinding of participants and key study personnel ensured, and unlikely that the blinding could have been broken; Incomplete blinding, such as blinding had to be uncovered because of characteristic side effect of intervention, but the outcome is not likely to be influenced by lack of blinding; No blinding, but the outcome is not likely to be influenced by lack of blinding.
Criteria for judgment of “High risk” of bias	Any one of the following: Single blinding of key study participants and personnel attempted, but likely that the blinding could have been broken, and the outcome is likely to be influenced by lack of blinding; No blinding or incomplete blinding, and the outcome is likely to be influenced by lack of blinding.
Criteria for judgment of “Unclear risk” of bias	Any one of the following: Insufficient information to permit judgment of ‘Low risk’ or ‘High risk’; The study did not address this outcome.

**Table A16:** Blinding of Outcome Assessment.

Detection Bias Due to Knowledge of the Allocated Interventions by Outcome Assessors	
Criteria for judgment of “Low risk” of bias	Any one of the following: Blinding of outcome assessment ensured, and unlikely that the blinding could have been broken; No blinding of outcome assessment, but the outcome measurement is not likely to be influenced by lack of blinding.
Criteria for judgment of “High risk” of bias	Any one of the following: Blinding of outcome assessment, but likely that the blinding could have been broken and the outcome measurement is likely to be influenced by lack of blinding; No blinding of outcome assessment, and the outcome measurement is likely to be influenced by lack of blinding.
Criteria for judgment of “Unclear risk” of bias	Any one of the following: Insufficient information to permit judgment of ‘Low risk’ or ‘High risk’; The study did not address this outcome.

**Table A17:** Incomplete Outcome Data.

Attrition Bias Due to Amount, Nature or Handling of Incomplete Outcome Data	
Criteria for judgment of “Low risk” of bias	Any one of the following: No missing outcome data; By checking the similarity between the remaining patients and loss to follow up patients, the reasons for missing outcome data unlikely to be related to preeclampsia such as migrating to another area; Missing outcome data balanced in numbers across intervention groups, with similar reasons for missing data across groups; For dichotomous outcome data, the proportion of missing outcomes compared with observed event risk not enough to have a clinically relevant impact on the intervention effect estimate; For continuous outcome data, plausible effect size (difference in means or standardized difference in means) among missing outcomes not enough to have a clinically relevant impact on observed effect size; Missing data have been imputed using appropriate methods.
Criteria for judgment of “High risk” of bias	Any one of the following: By checking the similarity between the remaining patients and loss to follow up patients, the reasons for missing outcome data likely to be related to preeclampsia such as diabetes mellitus, smoking status, BMI, with either imbalance in numbers or reasons for missing data across intervention groups; For dichotomous outcome data, the proportion of missing outcomes compared with observed event risk is enough to induce clinically relevant bias in intervention effect estimate; For continuous outcome data, plausible effect size (difference in means or standardized difference in means), among missing outcomes enough to induce clinically relevant bias in observed effect size; ‘As-treated’ analysis done with substantial departure of the intervention received from that assigned at randomization; Potentially inappropriate application of simple imputation.
Criteria for judgment of “Unclear risk” of bias	Any one of the following: Insufficient reporting of attrition/exclusions to permit judgement of ‘Low risk’ or ‘High risk’ (e.g., number randomized not stated, no reasons for missing data provided); The study did not address this outcome.

**Table A18:** Selective Reporting.

Reporting Bias Due to Selective Outcome Reporting	
Criteria for judgment of “Low risk” of bias	Any of the following: The study protocol is available and all of the study‘s pre-specified outcomes (preeclampsia, maternal and fetal outcomes) which are of interest in the review have been reported in the pre-specified way; The study protocol is not available, but it is clear that the published reports include all expected outcomes, including those that were pre-specified.
Criteria for judgment of “High risk” of bias	Any one of the following: Not all of the study’s pre-specified primary outcomes (preeclampsia) have been reported; One or more primary outcomes is reported using measurements, analysis methods or subsets of the data (e.g., early onset preeclampsia, late onset preeclampsia) that were not pre-specified; One or more reported primary outcomes were not pre-specified One or more outcomes of interest in the review are reported incompletely so that they cannot be entered in a meta-analysis; The study report fails to include results for a key outcome that would be expected to have been reported for such a study.
Criteria for judgment of “Unclear risk” of bias	Insufficient information to permit judgement of ‘Low risk’ or ‘High risk’. It is likely that the majority of studies will fall into this category.

**Table A19:** Other Bias.

Bias Due to Problems Not Covered Elsewhere in the Table	
Criteria for judgment of “Low risk” of bias	The study appears to be free of other sources of bias like baseline imbalance of important factors like obesity, or smoking by checking characteristics of participants between groups
Criteria for judgment of “High risk” of bias	There is at least one important risk of bias. For example, the study: Had a potential source of bias related to the specific study design used (e.g., problem in randomization, protocol violation, in cluster-randomized trials, there is loss of clusters (or) in cross-over trials, there is carry-over effect); or Had been claimed to have been fraudulent; or Had some other problem.
Criteria for judgment of “Unclear risk” of bias	There may be a risk of bias, but there is either: Insufficient information to assess whether an important risk of bias exists; or Insufficient rationale or evidence that an identified problem will introduce bias.
