# Supplementary figures and images for: Calcium and Vitamin D Supplementation for Prevention of Preeclampsia: A Systematic Review and Network Meta-Analysis
Source: Nutrients. 2017 Oct 18;9(10):1141. doi: 10.3390/nu9101141 (PMC5691757; doi:10.3390/nu9101141)

## Risk of Bias Assessment

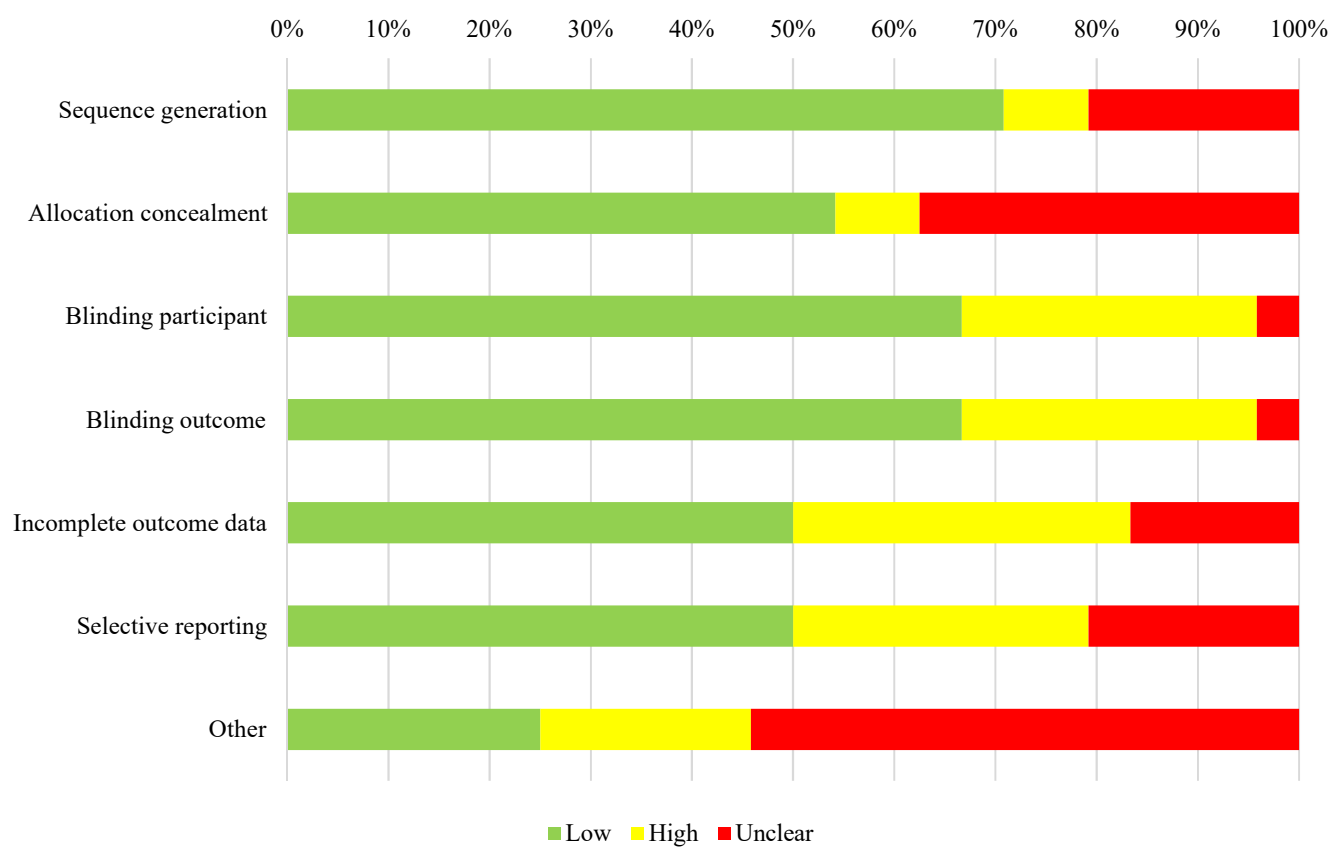

Supplement: Supplementary file 1 [file nutrients-09-01141-s001.zip › nutrients-226578supplementary/Figure S1.pdf]

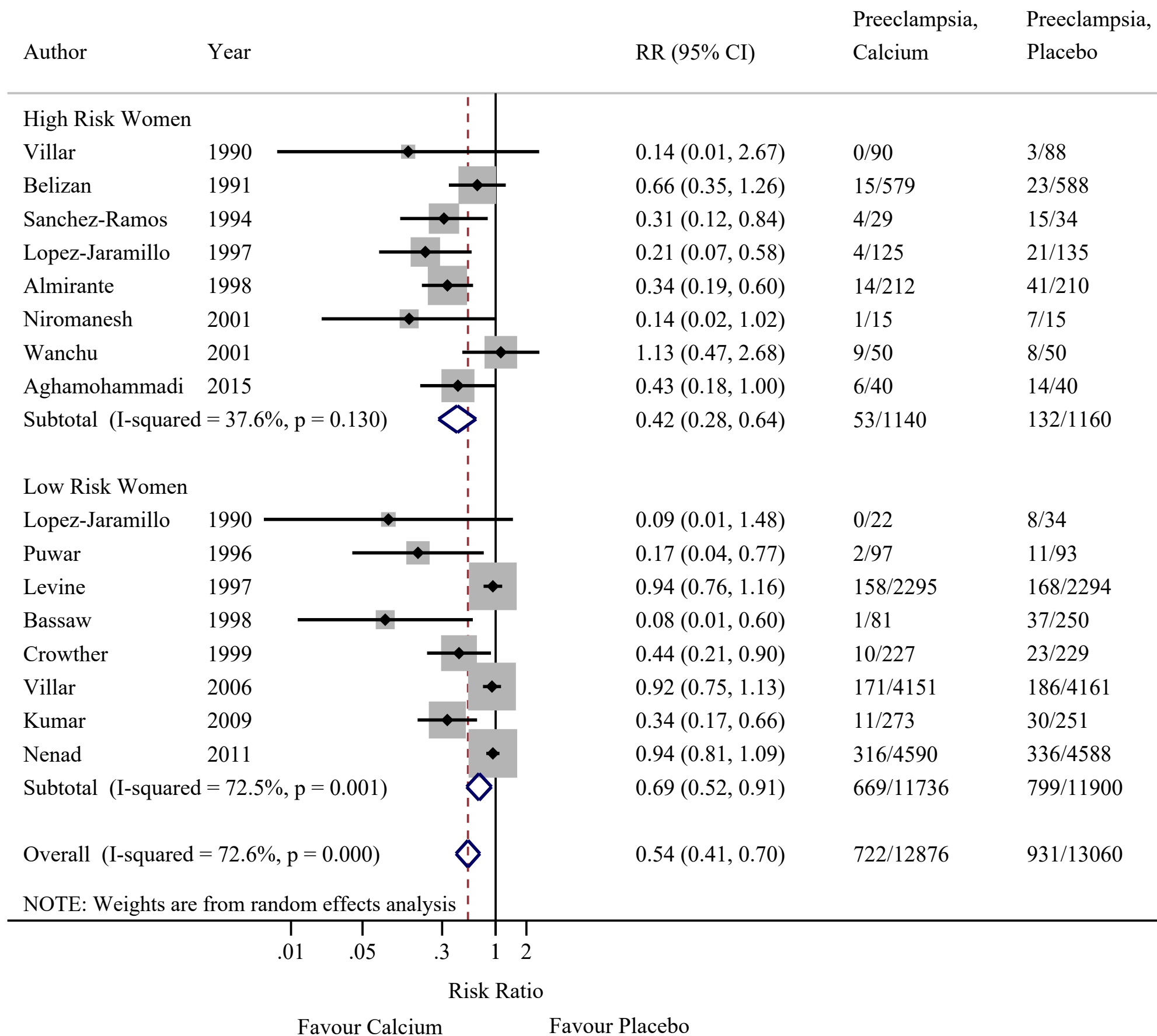

Supplement: Supplementary file 1 [file nutrients-09-01141-s001.zip › nutrients-226578supplementary/Figure S2a.pdf]

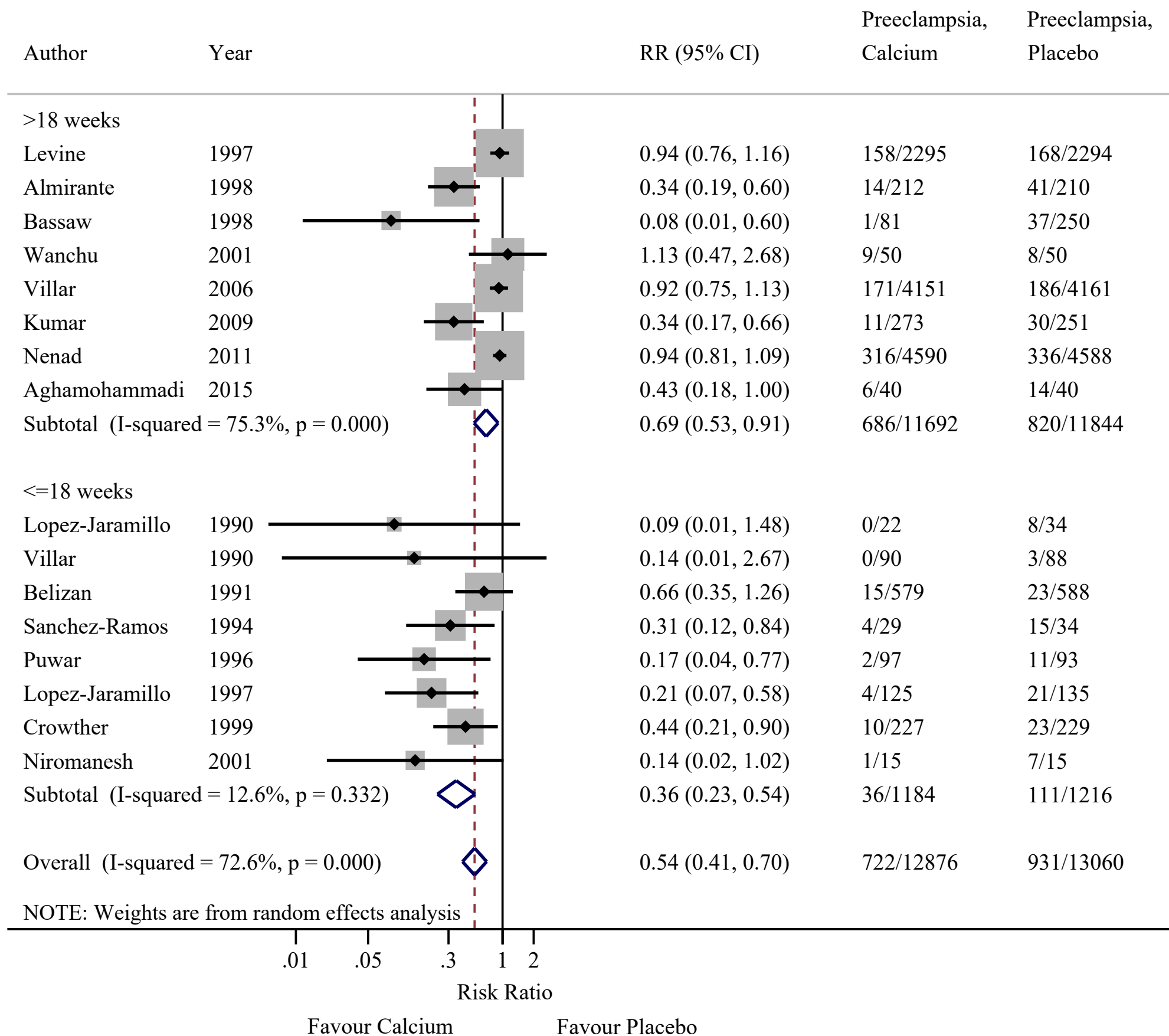

Supplement: Supplementary file 1 [file nutrients-09-01141-s001.zip › nutrients-226578supplementary/Figure S2b.pdf]

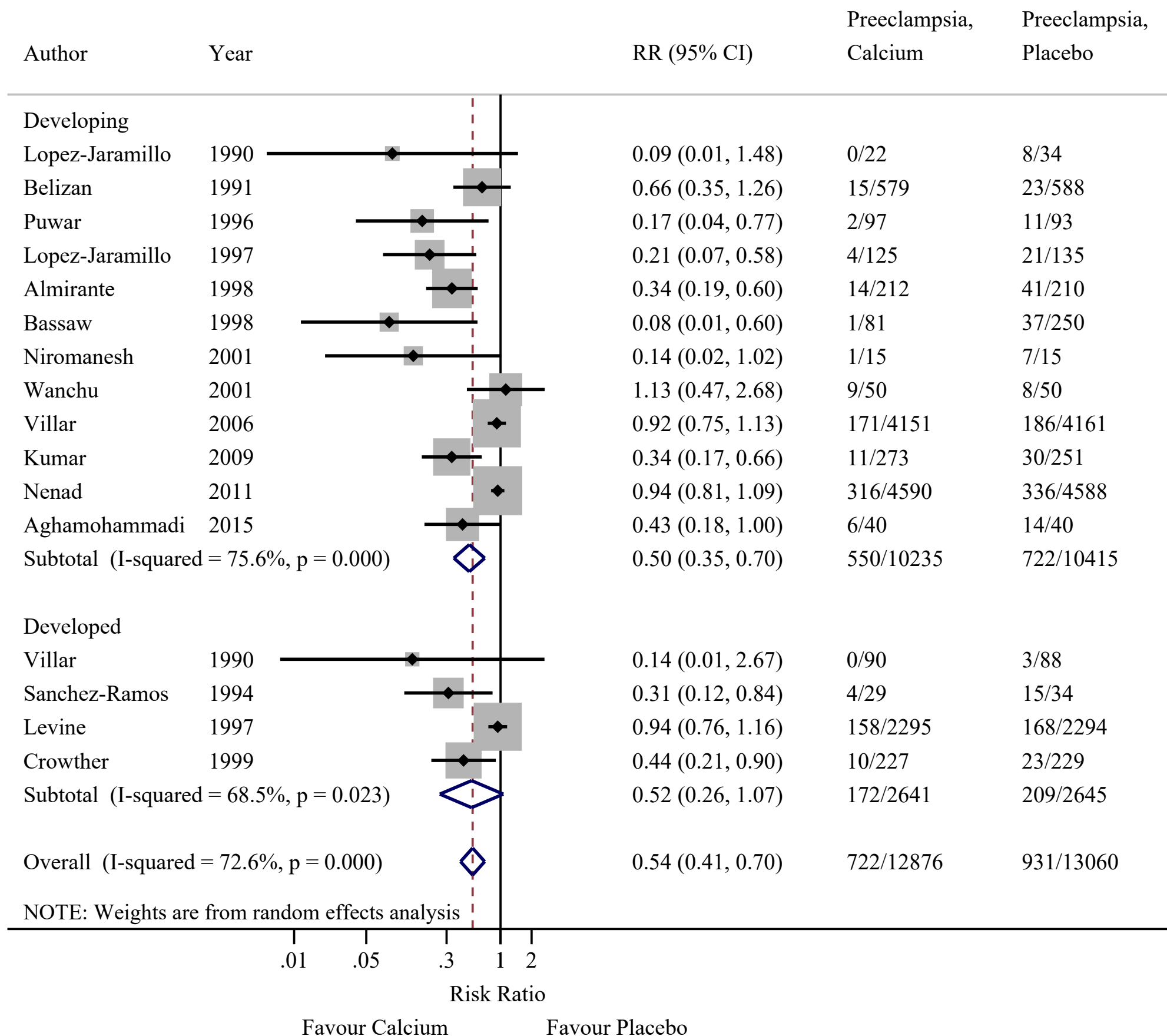

Supplement: Supplementary file 1 [file nutrients-09-01141-s001.zip › nutrients-226578supplementary/Figure S2c.pdf]

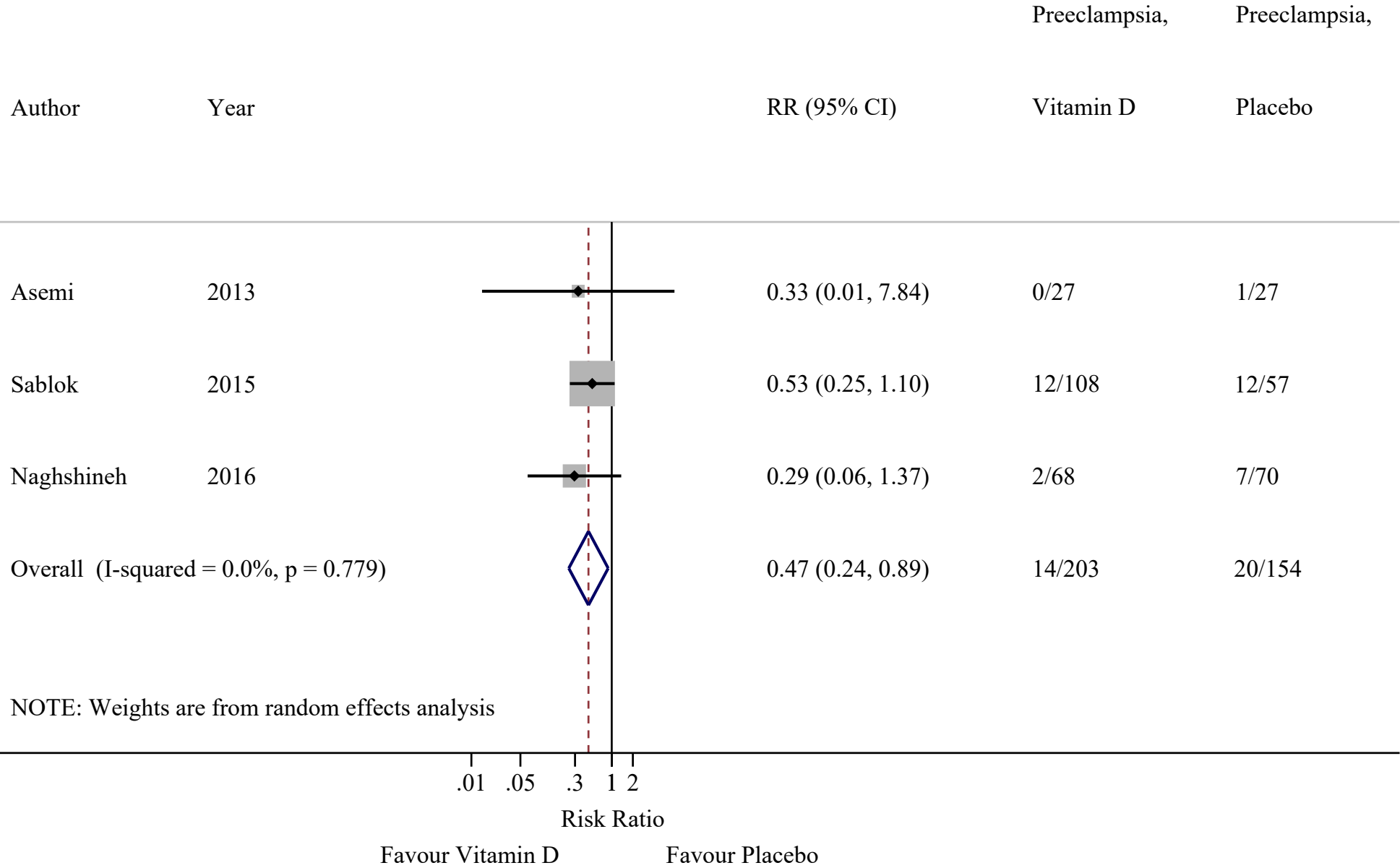

Supplement: Supplementary file 1 [file nutrients-09-01141-s001.zip › nutrients-226578supplementary/Figure S2d.pdf]

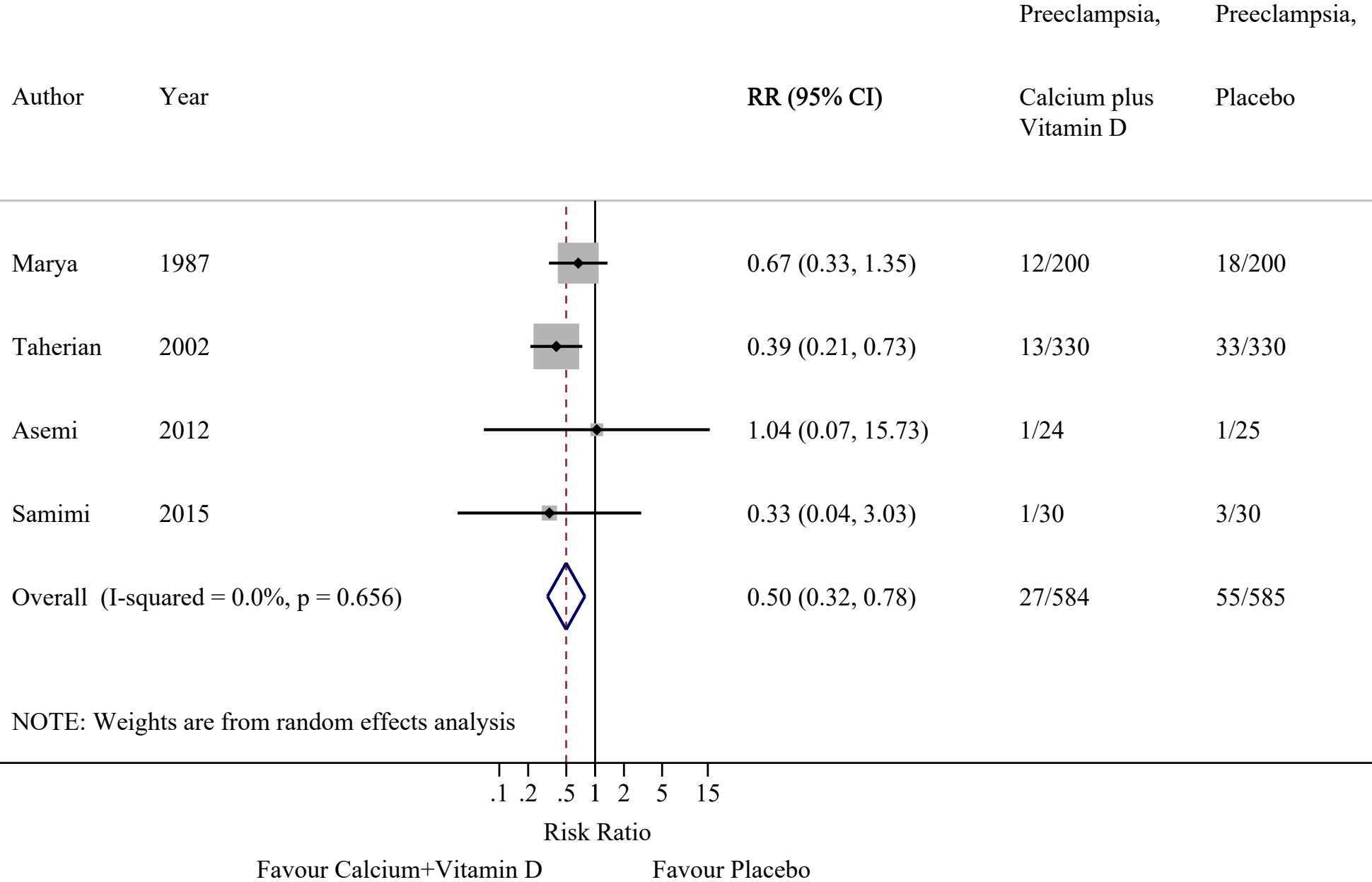

Supplement: Supplementary file 1 [file nutrients-09-01141-s001.zip › nutrients-226578supplementary/Figure S2e.pdf]

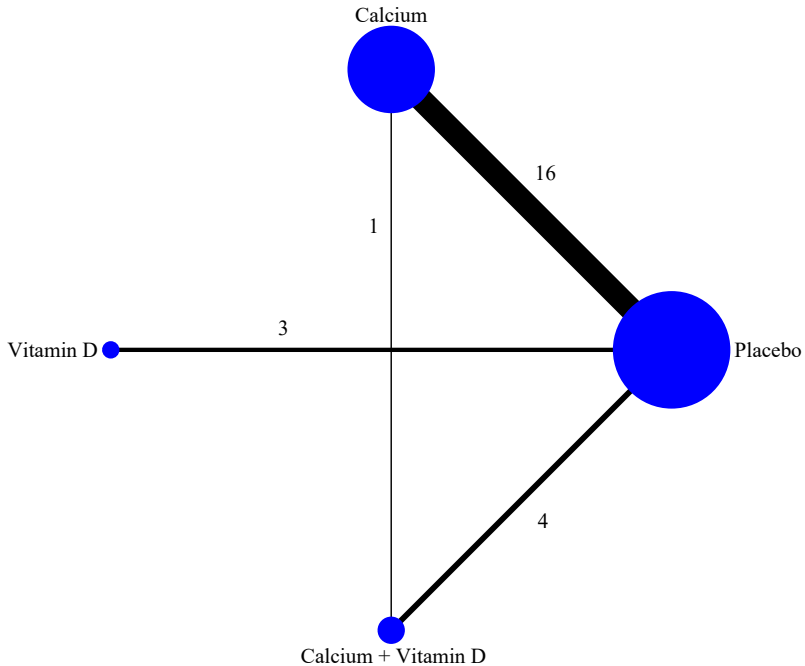

Supplement: Supplementary file 1 [file nutrients-09-01141-s001.zip › nutrients-226578supplementary/Figure S3.pdf]

Probabilities

Calcium

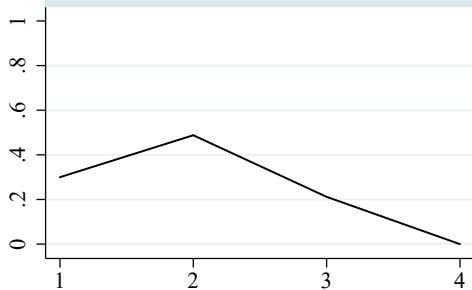

Calcium plus Vitamin D

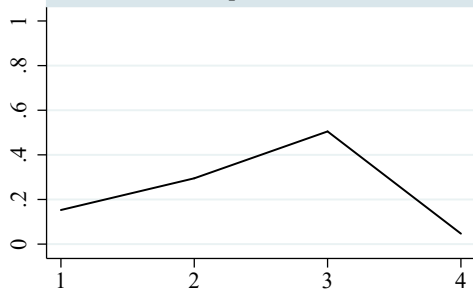

Placebo

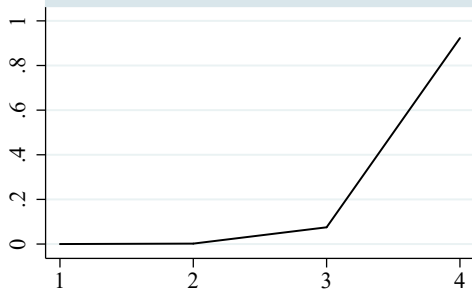

Vitamin D

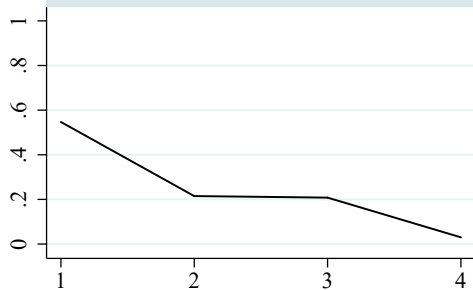

Rank

Supplement: Supplementary file 1 [file nutrients-09-01141-s001.zip › nutrients-226578supplementary/Figure S4.pdf]

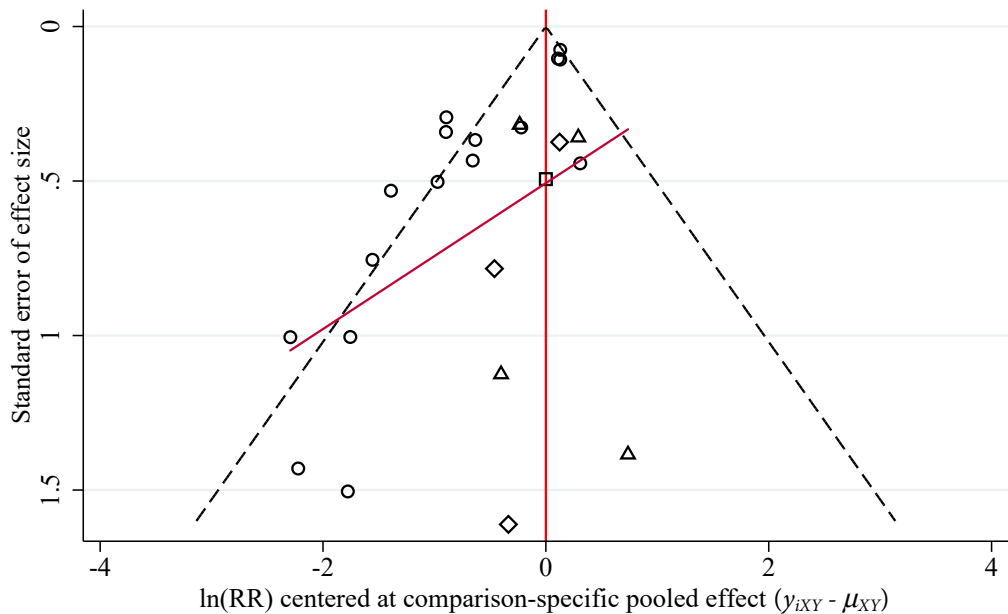

Supplement: Supplementary file 1 [file nutrients-09-01141-s001.zip › nutrients-226578supplementary/Figure S5.pdf]
