# Supplementary material for: Calcium and Vitamin D Supplementation for Prevention of Preeclampsia: A Systematic Review and Network Meta-Analysis
Source: Nutrients. 2017 Oct 18;9(10):1141. doi: 10.3390/nu9101141 (PMC5691757; doi:10.3390/nu9101141)
Supplement: Supplementary file 1 [file nutrients-09-01141-s001.zip › nutrients-226578supplementary/Supplementary.doc]

Supplementary: Calcium and Vitamin D Supplementation for Prevention of Preeclampsia: A Systematic Review and Network Meta-Analysis

Win Khaing ^1,2^, Sakda Arj-Ong Vallibhakara ^1,^*, Visasiri Tantrakul ^3^, Orawin Vallibhakara ^4^, Sasivimol Rattanasiri ^1^, Mark McEvoy ^5^, John Attia ^5^ and Ammarin Thakkinstian ^1^

Supplementary table legends

Table S1. Direct comparisons between supplementations on preeclampsia

Table S2. Direct comparisons between supplementations on gestational hypertension or pregnancy induced hypertension

Table S3. Risk of Bias Assessment

Table S4. Patient and methodological characteristics of included studies by treatment

**Table S1.** Direct comparisons between supplementations on preeclampsia

| **Author** | **Year** | **Type of pregnancy** | **n (intervention/ comparator)** | **No. events**  **(intervention/ comparator)** | **RR (95% CI)** |
| --- | --- | --- | --- | --- | --- |
| **Calcium versus placebo** | | | | | |
| Aghamohammadi [28] | 2015 | High risk | 40 / 40 | 6 / 14 | 0.43 (0.18, 1.00) |
| Almirante [29] | 1998 | High risk | 212 / 210 | 14 / 41 | 0.34 (0.19, 0.60) |
| Bassaw [30] | 1998 | Low Risk | 81 / 250 | 1 / 37 | 0.08 (0.01, 0.60) |
| Belizan [31] | 1991 | High risk | 579 / 588 | 15 / 23 | 0.66 (0.35, 1.26) |
| Crowther [32] | 1999 | Low Risk | 227 / 229 | 10 / 23 | 0.44 (0.21, 0.90) |
| Kumar [33] | 2009 | Low Risk | 273 / 251 | 11 / 30 | 0.34 (0.17, 0.66) |
| Levine [34] | 1997 | Low Risk | 2295 / 2294 | 158 / 168 | 0.94 (0.76, 1.16) |
| Lopez-Jaramillo [37] | 1990 | Low Risk | 22 / 34 | 0 / 8 | 0.09 (0.01, 1.48) |
| Lopez-Jaramillo [35] | 1997 | High risk | 125 / 135 | 4 / 21 | 0.21 (0.07, 0.58) |
| Nenad [38] | 2011 | Low Risk | 4590 / 4588 | 316 / 336 | 0.94 (0.81, 1.09) |
| Niromanesh [39] | 2001 | High risk | 15 / 15 | 1 / 7 | 0.14 (0.02, 1.02) |
| Puwar [40] | 1996 | Low Risk | 97 / 93 | 2 / 11 | 0.17 (0.04, 0.77) |
| Sanchez-Ramos [42] | 1994 | High risk | 29 / 34 | 4 / 15 | 0.31 (0.12, 0.84) |
| Villar [44] | 1990 | High risk | 90 / 88 | 0 / 3 | 0.14 (0.01, 2.67) |
| Villar [43] | 2006 | Low Risk | 4151 / 4161 | 171 / 186 | 0.92 (0.75, 1.13) |
| Wanchu [45] | 2001 | High risk | 50 / 50 | 9 / 8 | 1.13 (0.47, 2.68) |
| **Vitamin D versus placebo** | | | | | |
| Asemi [46] | 2013 | High risk | 27 / 27 | 0 / 1 | 0.33 (0.01, 7.84) |
| Naghshineh [47] | 2016 | High risk | 68 / 70 | 2 / 7 | 0.29 (0.06, 1.37) |
| Sablok [48] | 2015 | High risk | 108 / 57 | 12 / 12 | 0.53 (0.25, 1.10) |
| **Calcium plus vitamin D versus placebo** | | | | | |
| Asemi [49] | 2012 | High risk | 24 / 25 | 1 / 1 | 1.04 (0.07, 15.73) |
| Marya [50] | 1987 | Low Risk | 200 / 200 | 12 / 18 | 0.67 (0.33, 1.35) |
| Taherian [52] | 2002 | Low Risk | 330 / 330 | 13 / 33 | 0.39 (0.21, 0.73) |
| Samimi [51] | 2015 | High risk | 30 / 30 | 1 / 3 | 0.33 (0.04, 3.03) |
| **Calcium plus vitamin D versus calcium** | | | | | |
| Hossain [53] | 2014 | Low Risk | 89 / 86 | 6 / 10 | 0.58 (0.22, 1.53) |

**Table S2.** Direct comparisons between supplementations on gestational hypertension or pregnancy induced hypertension

| **Author** | **Year** | **Type of pregnancy** | **n (intervention/ comparator)** | **No. events**  **(intervention/ comparator)** | **RR (95% CI)** |
| --- | --- | --- | --- | --- | --- |
| **Calcium versus placebo** | | | | | |
| Bassaw [30] | 1998 | Low Risk | 81 / 250 | 0 / 26 | 0.06 (0.00, 0.94) |
| Belizan [31] | 1991 | High risk | 579 / 588 | 42 / 63 | 0.68 (0.47, 0.98) |
| Crowther [32] | 1999 | Low Risk | 227 / 229 | 34 / 38 | 0.90 (0.59, 1.38) |
| Levine [34] | 1997 | Low Risk | 2295 / 2294 | 351 / 397 | 0.88 (0.77, 1.01) |
| Lopez-Jaramillo [36] | 1989 | Low Risk | 49 / 43 | 2 / 12 | 0.15 (0.03, 0.62) |
| Lopez-Jaramillo [37] | 1990 | Low Risk | 22 / 34 | 3 / 24 | 0.19 (0.07, 0.57) |
| Nenad [38] | 2011 | Low Risk | 4590 / 4588 | 702 / 794 | 0.88 (0.81, 0.97) |
| Niromanesh [39] | 2001 | High risk | 15 / 15 | 9 / 4 | 2.25 (0.88, 5.73) |
| Puwar [40] | 1996 | Low Risk | 97 / 93 | 8 / 27 | 0.28 (0.14, 0.59) |
| Rogers [41] | 1999 | High risk | 144 / 75 | 21 / 18 | 0.61 (0.35, 1.07) |
| Sanchez-Ramos [42] | 1994 | High risk | 29 / 34 | 5 / 7 | 0.84 (0.30, 2.36) |
| Villar [11] | 1987 | Low Risk | 25 / 27 | 1 / 3 | 0.36 (0.04, 3.24) |
| Villar [44] | 1990 | High risk | 90 / 88 | 3 / 5 | 0.59 (0.14, 2.38) |
| Villar [43] | 2006 | Low Risk | 4151 / 4161 | 442 / 459 | 0.97 (0.85, 1.09) |
| **Calcium plus vitamin D versus calcium** | | | | | |
| Hossain [53] | 2014 | Low Risk | 89 / 86 | 11 / 7 | 1.63 (0.66, 4.00) |

**Table S3.** Risk of Bias Assessment

| **Authors (Year)** | **Sequence generation** | **Allocation concealment** | **Blinding of participants** | **Blinding of outcomes** | **Incomplete outcome data** | **Selective reporting** | **Other** |
| --- | --- | --- | --- | --- | --- | --- | --- |
| Aghamohammadi (2015) [28] | Unclear | Unclear | High | High | High | High | Unclear |
| Asemi (2012) [49] | Low | Low | Low | Low | High | Unclear | High |
| Asemi (2013) [46] | Low | Low | Low | Low | Low | Low | High |
| Bassaw (1998) [30] | Low | Low | Unclear | Unclear | High | Low | Low |
| Belizan (1991) [31] | Low | Low | Low | Low | Low | Low | Low |
| Crowther (1999) [32] | Low | Low | Low | Low | Low | Low | Unclear |
| Hossain (2014) [53] | Low | Unclear | High | High | Low | Low | Low |
| Kumar (2009) [33] | High | Low | Low | Low | Low | Low | Unclear |
| Levine (1997) [34] | Low | Low | Low | Low | Low | Unclear | Low |
| Lopez-Jaramillo (1989) [36] | Low | Unclear | Low | Low | Unclear | High | Unclear |
| Lopez-Jaramillo (1997) [35] | Low | Low | Low | Low | Unclear | Unclear | Unclear |
| Marya (1987) [50] | Unclear | Unclear | High | High | Unclear | Unclear | Unclear |
| Naghshineh (2016) [47] | Low | Low | High | High | High | High | Unclear |
| Niromanesh (2001) [39] | Unclear | Unclear | Low | Low | Low | Low | Unclear |
| Puwar (1996) [40] | Low | Unclear | Low | Low | Low | Low | Unclear |
| Rogers (1999) [41] | High | High | High | High | High | High | Unclear |
| Sablok (2015) [48] | Low | High | High | High | High | High | High |
| Samimi (2015) [51] | Low | Low | Low | Low | Low | Low | High |
| Sanchez-Ramos (1994) [42] | Low | Low | Low | Low | Low | Low | Low |
| Taherian (2002) [52] | Low | Unclear | Low | Low | Unclear | Unclear | High |
| Villar (1987) [11] | Unclear | Unclear | Low | Low | High | High | Unclear |
| Villar (1990) [44] | Low | Low | Low | Low | Low | Low | Unclear |
| Villar (2006) [43] | Low | Low | Low | Low | Low | Low | Low |
| Wanchu (2001) [45] | Unclear | Unclear | High | High | High | High | Unclear |

**Table S4.** Patient and methodological characteristics of included studies by treatment

| **Studies** | **Calcium Dosage**  **(mg)** | **Duration of Calcium Supplement**  **(weeks)** | **Mean GA at starting Calcium**  **(weeks)** | **Vitamin D Dosage**  **(IU)** | **Duration of Vitamin D Supplement**  **(weeks)** | **Mean GA at starting**  **Vitamin D**  **(weeks)** | **Mean Age**  **(years)** | **Mean GA at Enrolment**  **(weeks)** | **Mean GA at Delivery**  **(weeks)** | **BMI**  (kg/m^2^) |
| --- | --- | --- | --- | --- | --- | --- | --- | --- | --- | --- |
| Calcium vs Placebo | | | | | | | | | | |
| Lopez-Jaramillo 1990 | 2000 | 8 | 30 |  |  |  | 19.4 |  |  |  |
| Villar 1990 | 2000 | 15 | 24 |  |  |  | 16.3 | 23.6 | 38.6 |  |
| Belizan 1991 | 2000 | 15 | 23 |  |  |  | 23.7 | 20.8 |  |  |
| Sanchez-Ramos 1994 | 2000 | 12 | 26 |  |  |  | 18.4 | 24.4 |  |  |
| Puwar 1996 | 2000 | 18 | 20 |  |  |  | 21.9 | 18.1 | 37.5 |  |
| Levine 1997 | 1000 | 19 | 20 |  |  |  | 21 | 17.2 | 38.9 |  |
| Lopez-Jaramillo 1997 | 2000 | 19 | 20 |  |  |  | 16 | 20 | 39.1 |  |
| Almirante 1998 | 500 | 20 | 18 |  |  |  |  | 18 |  |  |
| Bassaw 1998 | 1200 | 20 | 19 |  |  |  | 27 |  | 38.6 |  |
| Crowther 1999 | 1800 | 18 | 20 |  |  |  | 24.7 | 18.4 |  | 26.6 |
| Niromanesh 2001 | 2000 | 9 | 30 |  |  |  | 23.2 | 29.7 | 38.6 |  |
| Wanchu 2001 | 2000 | 18 | 20 |  |  |  |  | 14.2 |  |  |
| Villar 2006 | 1500 | 22 | 16 |  |  |  | 22.7 | 15.1 |  | 21.9 |
| Kumar 2009 | 2000 | 20 | 18 |  |  |  | 21.9 | 17.8 | 38.4 | 23.4 |
| Nenad 2011 | 2000 | 19 | 19 |  |  |  |  | 18.5 |  |  |
| Aghamohammadi 2015 | 1000 | 19 | 19 |  |  |  | 37.2 |  |  | 26.8 |
|  |  |  |  |  |  |  |  |  |  |  |
| Mean | 1687.5 | 16.9 | 21.4 |  |  |  | 22.6 | 19.7 | 38.5 | 24.7 |
| Vitamin D vs Placebo | | | | | | | | | | |
| Asemi 2013 |  |  |  | 50000 | 6 | 26 | 17.4 | 26 |  | 30.8 |
| Sablok 2015 |  |  |  | 120000 |  |  |  |  |  |  |
| Naghshineh 2016 |  |  |  | 600 | 21 | 16 | 25 |  | 37.4 |  |
|  |  |  |  |  |  |  |  |  |  |  |
| Mean |  |  |  | 56866.7 | 13.5 | 21.0 | 21.2 | 26.0 | 37.4 | 30.8 |
| Calcium + Vitamin D vs Placebo | | | | | | | | | | |
| Marya 1987 | 375 | 16 | 22 | 1200 | 18 | 22 |  | 22 |  |  |
| Taherian 2002 | 500 | 19 | 20 | 200 | 18 | 20 | 21.6 | 20 | 38.8 | 22.6 |
| Asemi 2012 | 600 | 13 | 25 | 200 | 9 | 25 | 24.9 |  |  | 27.6 |
| Samimi 2015 | 1000 | 18 | 20 | 3600 | 12 | 20 | 27.2 |  |  | 26.5 |
|  |  |  |  |  |  |  |  |  |  |  |
| Mean | 618.8 | 16.5 | 21.8 | 1300.0 | 14.3 | 21.8 | 24.6 | 21.0 | 38.8 | 25.6 |
|  |  |  |  |  |  |  |  |  |  |  |
| Calcium + Vitamin D vs Calcium | | | | | | | | | | |
| Hossain 2014 | 600 | 18 | 20 | 4000 | 18 | 20 | 25.6 | 20 | 37.6 | 23.6 |
|  |  |  |  |  |  |  |  |  |  |  |
| Mean | 600 | 18 | 20 | 4000 | 18 | 20 | 25.6 | 20 | 37.6 | 23.6 |

GA = Gestational Age (weeks), BMI = Mean Body Mass Index (kg/m^2^)

Supplementary Figure legends

Figure S1. Risk of bias assessment

Figure S2. Direct comparisons of supplementations on preeclampsia

a) Calcium vs placebo by participants
b) Calcium vs placebo by duration of calcium supplementation
c) Calcium vs placebo by country setting
d) Vitamin D vs placebo
e) Calcium plus vitamin D vs placebo

Figure S3. Network of all possible interventions for prevention of preeclampsia

Figure S4. Rankogram of calcium, vitamin D, calcium plus vitamin D and placebo effects on preeclampsia

Figure S5. Comparison-adjusted funnel plot for the network meta-analysis
